# Synthesis of methyldopa-copper nanoparticles with laccase mimics activity for colorimetric detection of norepinephrine

**DOI:** 10.1038/s41598-026-61978-6

**Published:** 2026-07-17

**Authors:** Aya A. Mouhamed, Amr M. Mahmoud, Jeffrey G. Bell, Ola G. Hussein

**Affiliations:** 1 Department of Pharmaceutical Analytical Chemistry, Faculty of Pharmacy, Cairo University, Kasr El-Aini Street, Cairo, ET-11562 Egypt; 2 Department of Chemistry, Washington State University, Pullman, WA 99164 USA; 3 The Gene and Linda Voiland School of Chemical Engineering and Bioengineering, Washington State University, Pullman, 99164 WA USA; 4 Department of Pharmaceutical Chemistry, Faculty of Pharmacy, Future University in Egypt, Cairo, 11835 Egypt

**Keywords:** Biocatalysis, Copper-based nanozyme, Enzyme kinetics, Laccase mimic, Methyldopa metal-organic, Norepinephrine oxidation, Biochemistry, Chemistry, Environmental sciences, Nanoscience and technology

## Abstract

Copper-based nanozymes are increasingly used as artificial laccases, yet most rely on simple catechol ligands such as dopamine or gallic acid which provide limited control over metal coordination geometry and stability. In this work, we introduce methyldopa as a biomimetic bidentate ligand whose dual catechol-amine functionality closely reproduces the coordination environment of natural multicopper oxidases. This molecular design enables electronic tuning of the Cu(I)/Cu(II) redox cycle resulting in enhanced substrate affinity and exceptional thermal resilience. The prepared polymethyldopa-Cu nanoparticles (PMD-Cu NPs) exhibit robust laccase-like activity under near-physiological conditions catalyzing the oxidation of norepinephrine without additional oxidants. Beyond catalysis, the oxidation process provides an intrinsic colorimetric signal allowing simultaneous quantification and degradation of catecholamine pollutants as pyrogallol. Kinetic analysis (*K*_*m*_= 45 µM; *V*_*max*_= 5.0 µM/ min) demonstrates excellent catalytic performance and favorable substrate affinity compared with previously reported Cu-catechol nanozymes. By coupling strong Cu-ligand chelation with redox-active molecular design, methyldopa permits the construction of durable multifunctional nanozymes. This approach establishes a pathway toward tunable laccase mimics for biosensing, green chemistry, and environmental remediation. In addition, the sustainability and analytical efficiency of the developed platform were evaluated using the newly introduced EPPI metric alongside BAGI tool confirming its eco-friendly nature, practical applicability, and strong analytical performance.

**Supplementary Information:**

The online version contains supplementary material available at 10.1038/s41598-026-61978-6.

## Introduction

Enzyme-inspired nanomaterials, or nanozymes have emerged as powerful catalytic platforms capable of mimicking and in some cases surpassing the reactivity of biological enzymes^1–5^. Unlike natural enzymes, which are often expensive to isolate, can quickly degrade, and require specific storage conditions, nanozymes offer streamlined fabrication processes, tunable functionality, and improved robustness^6–13^. The rapid evolution of nanozyme-based sensing has been accompanied by the development of portable analytical platforms and advanced recognition approaches, enabling simple, rapid, and reliable analyses with improved sensitivity, selectivity, and applicability in real-world samples^14,15^.

Among these, copper-based laccase mimics are of particular interest because they can oxidize phenolic and catechol substrates via molecular oxygen promoting sustainable applications in biosensing, pollutant degradation, and green chemistry^16–18^. The absence of harmful intermediates such as hydrogen peroxide makes laccase-mimics attractive for green chemistry particularly for degrading aromatic pollutants in environmental systems^19–21^. However, despite the rapid growth in nanozyme research, most reported Cu-based systems employ simple catechol derivative ligands such as dopamine, gallic acid, or tannic acid which offer only oxygen-donor coordination^22–27^. This chemical design limits control over the copper redox environment which can lead to unstable Cu(I)/Cu(II) cycling, poor durability, and inefficient mimicry of multicopper active sites present in natural laccase^26,28^. Hybrid materials incorporating copper ions within organic or metal-organic frameworks have been designed to reproduce laccase-like redox behavior often using nitrogen or sulfur donor ligands to reproduce enzymatic coordination environments. Despite this progress, more efficient, robust, and cost-effective laccase-like catalysts are needed^29,30^.

To address these constraints, we introduce methyldopa as a dual-function catechol-amine ligand for constructing a new class of polymethyldopa-copper nanoparticles (PMD-Cu NPs)^31–36^. Methyldopa’s unique chemical structure provides both catechol (O-donor) and amine (N-donor) coordination sites resembling the histidine-catechol environment that stabilizes Cu centers in natural multicopper oxidases^37^. This dual coordination enables improved Cu-ligand chelation, enhanced redox reversibility, and a more stable Cu(I)/Cu(II) cycling^38–40^. Beyond stability, the electron-rich catechol moiety facilitates rapid substrate oxidation while the amine substituent contributes to structural rigidity and prevents aggregation. In addition to their catalytic applications, laccase-inspired nanomaterials have been exploited towards the design of colorimetric analytical sensors for the analysis of diverse analytes such as norepinephrine (NE)^24,41,42^. These nanozymes promote the oxidation of NE, in the presence of molecular oxygen resulting in the generation of a colored product that can be quantified via UV-VIS analysis. NE, a catecholamine, poses environmental risks when released into water systems via pharmaceutical waste or metabolic byproducts^43,44^. Its controlled degradation is therefore of significant interest in environmental remediation. Herein, PMD-Cu NPs were developed to mimic laccase-like activity and applied towards the catalytic oxidation of NE. The PMD-Cu NPs efficiently catalyze the oxidation of NE by mediating electron transfer from its catechol moiety to dissolved oxygen resulting in the generation of norepinephrine-o-quinone along with the release of two protons under mild aqueous conditions. Rather than modifying natural enzymes, PMD-Cu NPs demonstrate how rationally designed nanoarchitectures can perform targeted biochemical transformations with enhanced efficiency and reusability. This shift toward engineered catalytic platforms represents a promising strategy for advancing enzyme-inspired applications.

By employing methyldopa as a rationally chosen ligand, this work advances the molecular design principles of laccase-like nanozymes. The resulting PMD-Cu NPs combine high substrate affinity with remarkable thermal and pH stability supporting both oxidative degradation and colorimetric quantification of phenolic analytes such as NE. Additionally, its structure promotes regeneration and reusability without significant loss of catalytic activity. This dual functionality bridges the gap between analytical sensing and environmental remediation demonstrating how targeted ligand design can yield multifunctional enzyme-like catalysts. The design strategy reflects a broader trend in nanozyme development where the goal is not only imitating the activity of natural enzymes but to exceed the limitations exhibited by their biological counterparts. These insights broaden the approaches for constructing copper-based nanozymes while also establishing a generalizable route for tuning the redox chemistry of biomimetric catalytic materials.

## Experimental

### Analytical equipment and software tools

The physical and elemental characteristics of the prepared nanomaterials were examined using scanning electron microscopy (SEM) along with energy-dispersive X-ray spectroscopy (EDX) both performed on a Quanta FEG-250 system and High-resolution transmission electron microscopy (HRTEM) was performed using a JEM-2100 HRT instrument (JEOL, Japan). A PrO-Research benchtop refrigerated centrifuge (Centurion Scientific Ltd., UK) was employed for all centrifugation procedures. The instrument offers a variable speed range from 500 to 15,000 rpm. Functional group analysis and chemical bonding insights were achieved through FT-IR spectroscopy using a Shimadzu IR 435 device. Structural information regarding crystallinity and phase composition was collected via X-ray diffraction (XRD) employing the Rigaku Smart Lab system. The thermal stability of the PMD-Cu NPs was assessed through thermogravimetric analysis (TGA) using a Shimadzu TGA-50 instrument (Kyoto, Japan). To evaluate particle size distribution, dynamic light scattering (DLS) analysis was conducted using a Zetasizer Nano-ZS from Malvern Instruments (UK). X-ray photoelectron spectroscopy was performed using an XPS instrument equipped with a monochromatic Al Kα radiation source (1486.6 eV) to investigate the surface elemental composition and electronic structure of the sample. Optical absorbance data were obtained using a SHIMADZU UV-1900i spectrophotometer (Kyoto, Japan) equipped with a dual-beam setup and controlled through UV Probe software (version 2.43). The measurements were conducted with 1 cm quartz cuvette using a 1 nm spectral resolution and a scanning speed of 2800 nm per minute.

### Materials and reagents

All required materials for synthesis and analysis were sourced from certified suppliers and used without any additional refinement. Methyldopa (98%), copper (II) sulfate anhydrous (99%), and norepinephrine (NE) (98%) were procured from Sigma-Aldrich (USA). Sodium hydroxide (98%), hydrochloric acid (37% w/w), and sodium dihydrogen phosphate (99%) were supplied by Bio-chem (Egypt). For the preparation of buffer solutions, a 0.02 M sodium dihydrogen phosphate solution was used as the base and its pH was carefully adjusted to 6.0 by gradual titration with NaOH under continuous stirring to ensure uniformity using pH glass electrode (Jenway, UK). All reagents used were of analytical purity and handled according to standard laboratory protocols.

### Standard solutions

#### Preparation of norepinephrine standard solutions

Initial stock solution of NE was prepared at a concentration of 1.0 mg/mL by dissolving the appropriate amount in distilled water.

### Synthesis of PMD-Cu NPs

The PMD-Cu NPs were synthesized following synthetic an established literature procedure to ensure reproducibility and structural integrity^45^. To prepare the PMD-Cu NPs, two solutions were first prepared separately: one containing methyldopa at a concentration of 0.5 mg/mL and another containing copper (II) sulfate pentahydrate using a 2:1 mass ratio of methyldopa to copper. Equal volumes of these two solutions were then combined and the pH of the mixture was carefully adjusted to 7.06 ± 0.05 using a calibrated digital pH meter (Jenway, UK). The system was stirred continuously at room temperature for 24 h to enable effective coordination between the methyldopa units and copper ions. Under these near-neutral conditions and prolonged reaction time, methyldopa undergoes spontaneous oxidative polymerization while simultaneously coordinating with Cu^2+^ ions following the previously reported synthesis of Cu-chelated polydopamine nanozymes^45^. Therefore, the resulting material was designated as polymethyldopa-Cu nanoparticles (PMD-Cu NPs).

Following the reaction, the resulting nanoparticles were separated by centrifuging the solution at 12,000 rpm for 10 min. The solid product was then washed three times with deionized water to ensure removal of residual ions and unbound components.

### Structural and analytical evaluation of PMD-Cu NPs

To investigate the physical and chemical properties of the synthesized PMD-Cu NPs, a suite of characterization tools was employed. Morphological examination was carried out using scanning electron microscopy and High-resolution transmission electron microscopy (HRTEM) to observe particle form and surface structure. Elemental mapping and composition were determined through EDX analysis confirming the integration of copper and methyldopa components. FTIR spectroscopy offered insight into the bonding environment within the framework identifying functional groups involved in metal-ligand coordination. Crystallinity and phase purity were assessed via XRD revealing structural features of the synthesized material. XPS measurements verified the presence of copper and clarified its oxidation states offering direct insight into the surface chemistry and confirming the successful formation of copper-coordinated nanozymes. Thermal behavior including decomposition patterns was evaluated through thermogravimetric analysis under controlled heating conditions. UV-visible spectroscopy was also applied to examine the electronic transitions that may influence redox behavior. This multi-technique assessment was essential to establish the material’s characteristics and its potential as a stable and functional nanozyme.

### Investigation of oxidative catalysis by PMD-Cu NPs

The ability of the synthesized PMD-Cu NPs to exhibit laccase-like oxidative behavior was examined using NE as the model substrate. A total reaction volume of 3.0 mL was prepared by mixing 10.0 µL of Cu-Methyldopa suspension (1.0 mg/mL) with 24.0 µL of NE solution and the volume was completed by phosphate buffer (pH adjusted to 6.0). This mixture was incubated at 40 °C for a duration of 10 min to allow sufficient catalytic oxidation to occur. Post-incubation, UV-visible absorbance readings were collected to monitor product formation. To confirm the specific catalytic role of the PMD-Cu NPs a parallel test was conducted under identical conditions excluding the catalyst. All experiments were performed in triplicate (*n*= 3) and the results are expressed as the mean ± standard deviation (SD).

### Influence of pH on the catalytic efficiency of PMD-Cu NPs

The role of pH in modulating the catalytic function of PMD-Cu NPs was evaluated by initiating oxidative reactions under controlled buffer conditions. Equal volumes (50.0 µL each) of PMD-Cu NPs dispersion (1.0 mg/mL) and NE solution and the total volume was adjusted to 3.0 mL using the same phosphate buffer (pH 6.0). The reaction was allowed to be incubated at 40 °C for 30 min. Following incubation, absorbance was measured to assess the extent of substrate oxidation thereby reflecting the pH sensitivity of nanozyme.

### Temperature response of PMD-Cu NPs during substrate oxidation

To explore the thermal resilience and activity window of PMD-Cu NPs, the oxidation of NE was carried out across a range of temperatures. The catalyst (50.0 µL, 1.0 mg/mL) and NE (100.0 µL) were combined in a 700.0 µL aliquot of phosphate buffer at pH 6.0. The assembled mixtures were incubated for 30 min at varying temperatures from 30 °C to 90 °C. UV-visible spectroscopy was used post-incubation to evaluate product formation.

### Monitoring oxidation rate of norepinephrine

To track the progression of NE oxidation catalyzed by PMD-Cu NPs, a reaction system containing 50.0 µL of the catalyst (1.0 mg/mL) and 50.0 µL of NE was prepared and completed to volume using pH 6.0 phosphate buffer. The reaction proceeded at 40 °C and aliquots were collected every 5 min over a 60-minute period. Each sample was immediately analyzed via UV-Vis spectrophotometry to monitor changes in absorbance thereby mapping the reaction kinetics over time.

### Quantitative detection of norepinephrine using PMD-Cu NPs

To establish the relationship between NE concentration and optical signal, a colorimetric assay was conducted using variable volumes of NE stock solution (50.0 µM) combined with 50.0 µL of PMD-Cu NPs (1.0 mg/mL). The volume in each case was completed with phosphate buffer (pH 6.0) generating final concentrations in the range of 4.5–100 µM. After 30 min of incubation at 40 °C the absorbance values of the reaction mixtures were measured. The resulting calibration curve confirmed the suitability of the nanozyme for NE quantification.

### Enzyme kinetic profiling of PMD-Cu NPs with norepinephrine

Kinetic behavior of PMD-Cu NPs in catalyzing NE oxidation was examined under conditions of fixed catalyst concentration (1.0 mg/mL) and varying substrate concentrations (0.1 to 50 mM). Reaction rates were determined from absorbance data and analyzed by fitting the values to the Michaelis-Menten model. The resulting kinetic constants (*K*_*m*_ and *V*_*max*_) provided insights into the binding affinity and catalytic efficiency of the nanozyme system under physiological pH.

### Investigation of PMD-Cu NPs reusability in the oxidative catalysis of NE

To evaluate the catalyst stability and practical applicability, we have performed recycling experiments to confirm whether the prepared PMD-Cu NPs can be reused. For the recyclability test, PMD-Cu NPs (1.0 mg/mL) and NE (1.0 mM) were taken in a 2 mL Eppendorf tube and after 30 min incubation at 40 °C, the reaction mixture was centrifuged at 4000 rpm for 15 min. Then, the UV-Vis spectra were recorded for the supernatant solution. Subsequently, PMD-Cu NPs was washed three times with distilled water and a similar catalytic reaction was performed.

### Evaluation of sustainability and analytical performance

The environmental impact and analytical effectiveness of the proposed sensing methodology were comprehensively investigated using the recently introduced EPPI metric together with the Blue Applicability Grade Index (BAGI). The EPPI approach was utilized to estimate the overall balance between ecological sustainability and methodological efficiency. Meanwhile, BAGI analysis provided insight into the operational feasibility and practical utility of the developed procedure. The collective outcomes of these evaluation tools confirm its eco-friendly nature, practical applicability, and strong analytical performance.

## Results and discussion

### Thermal stability and particle size analysis

**Fig. 1:**
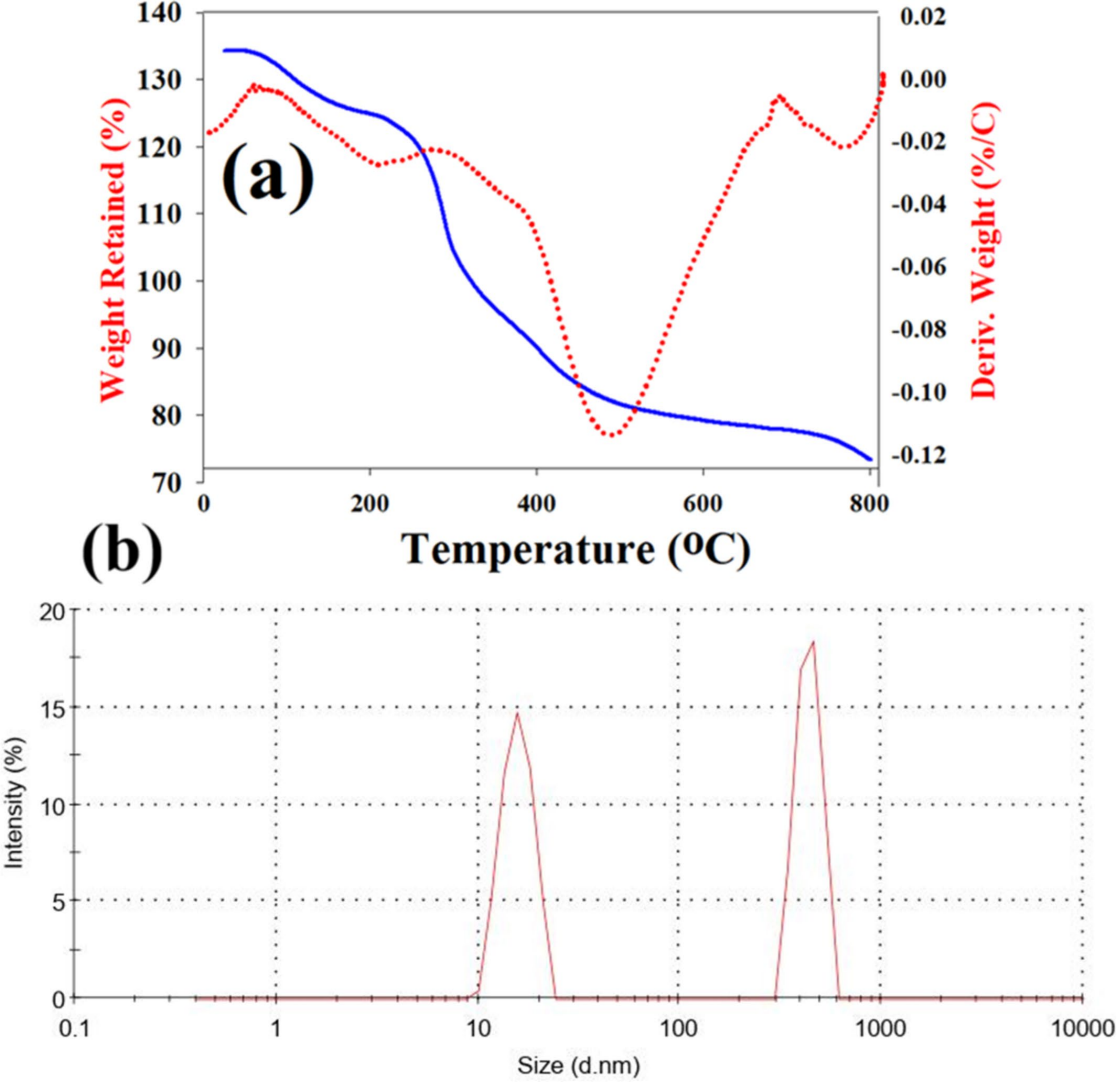
(**a**) TGA analysis and (**b**) Dynamic light scattering (DLS) spectrum of PMD-Cu NPs.

The thermal behavior of the methyldopa-Cu nanoparticles (PMD-Cu NPs) was examined by thermogravimetric analysis (TGA) and derivative thermogravimetry (DTG) as shown in Fig. 1a. The TGA curve (blue) indicates an initial weight loss below 150 °C attributed to the removal of physically adsorbed water and residual solvent. A major weight loss occurs between 200 and 450 °C corresponding to the decomposition of the methyldopa organic layer coordinated to the Cu surface. The DTG peak near 400 °C confirms the degradation of the capping molecules including aromatic and carboxylate functionalities. Beyond 450 °C, the weight stabilizes with only a minor decline up to 800 °C indicative of a thermally stable inorganic residue, presumably CuO formed through oxidation of metallic Cu during heating. The overall weight retention (75–80%) suggests a high inorganic (Cu-based) fraction confirming successful nanoparticle formation with substantial metal content. Dynamic light scattering (DLS) was performed to evaluate the hydrodynamic characteristics of the synthesized PMD-Cu nanoparticles in aqueous medium. As shown in Fig. 1b, the nanoparticles exhibited a Z-average hydrodynamic diameter of 419.5 nm with a polydispersity index (PdI) of 0.468. The intensity-based size distribution revealed two particle populations centered at approximately 15.9 and 435.5 nm indicating the presence of multiple hydrodynamic populations in the colloidal suspension. The corresponding zeta potential analysis (**Fig. ****S1**) showed a surface potential of -43.0 mV demonstrating a highly negatively charged particle surface and suggesting good electrostatic colloidal stability. The complete DLS and zeta potential reports are provided in the Supplementary Information.

### Morphological, compositional, and structural characterization

**Fig. 2:**
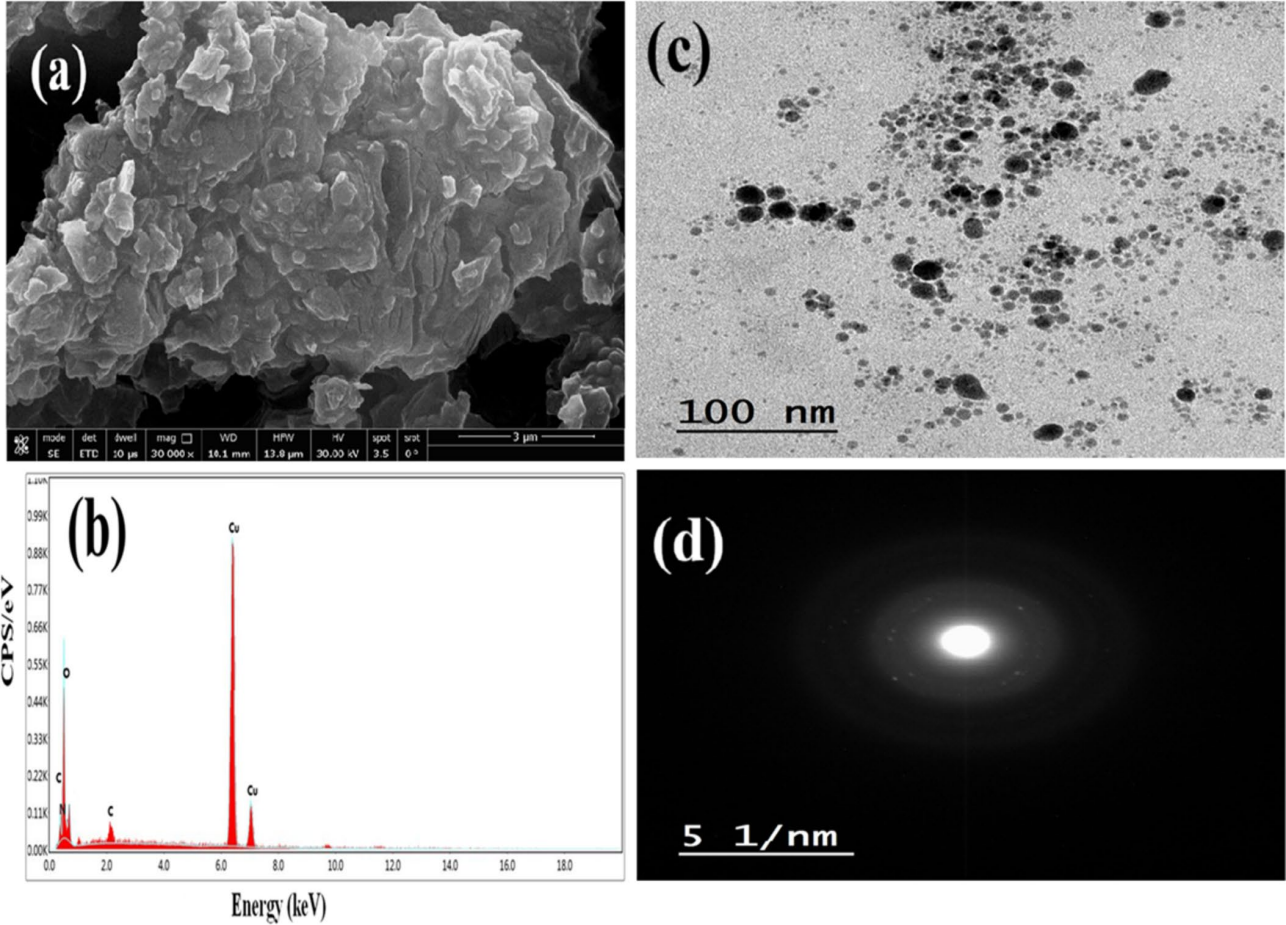
(**a**) The SEM image, (**b**) EDX spectrum, (**c**) TEM image displaying uniformly distributed PMD-Cu NPs with nanoscale dimensions, and (**d**) SAED pattern showing distinct concentric diffraction rings confirming the polycrystalline nature of the PMD-Cu NPs.

The morphology and composition of PMD-Cu NPs were characterized using SEM, EDX, TEM, and SAED as presented in Fig. 2. The SEM image (Fig. 2a) reveals aggregated plate-like structures with rough surfaces and irregular boundaries typical of metal-organic hybrid materials. This morphology arises from the partial stacking or clustering of methyldopa-capped nanoparticles during the drying process. The EDX spectrum (Fig. 2b) displays intense Cu peaks along with C, N, and O signals corresponding to the methyldopa ligand confirming the successful coordination of the organic capping molecule to the metal surface. The absence of additional peaks indicates high purity and efficient reduction of Cu ions. TEM imaging (Fig. 2c) shows well-dispersed nearly spherical nanoparticles with average diameters ranging from 50 to 80 nm consistent with DLS data. The relatively uniform contrast and low degree of agglomeration highlight the strong interaction between Cu nanoparticles and methyldopa molecules which hinder particles aggregation. The selected area electron diffraction (SAED) pattern (Fig. 2d) exhibits concentric diffraction rings indexed to the (111), (200), and (220) planes characteristic of the face-centered cubic (fcc) Cu phase confirming the polycrystalline nature of the crystalline nanoparticle core. FTIR analysis (**Fig. S2a**) revealed noticeable shifts in the characteristic vibrational bands of methyldopa after coordination with Cu ions indicating successful metal-ligand interactions. The broad O–H stretching band around 3400 cm^[-1^ together with the bands assigned to the C = O and N–H functional groups exhibited shifts in both position and intensity relative to free methyldopa suggesting the involvement of the hydroxyl and amino groups in copper coordination Similar spectral changes have been widely reported for copper–catechol coordination systems. It should be noted that the direct Cu–N stretching vibration is generally observed in the far-infrared (FIR) region (approximately 250–450 cm^[-1^ which lies outside the spectral range investigated in the present FTIR measurements. Therefore, the coordination between methyldopa and Cu ions was inferred from the systematic changes in the ligand vibrational bands and further corroborated by the complementary XPS, XRD, and EDX characterization results^46^. XRD analysis further supports complex formation and structural evolution of the PMD-Cu NPs. The diffraction pattern shows a combination of broad features and distinct peaks indicating partial crystallinity. Reflections observed between 20^o^ and 40^o^ (2θ) are characteristic of copper-containing crystalline domains suggesting localized ordered regions within the hybrid structure. The absence of diffraction peaks corresponding to free methyldopa confirms complete coordination with Cu ions and ligand structural modification. Additionally, low-angle diffraction features commonly observed in metal-organic systems likely arise from periodic coordination motifs or short-range ordering of the polymeric framework^47^(**Fig. S2b**). XPS analysis further substantiated the surface chemical composition and oxidation states of copper within the PMD-Cu NPs. Decomposition of the Cu 2p spectrum revealed dominant Cu^2+^ contributions at binding energies of 934.8 eV (Cu 2p_3/2_) and 954.6 eV (Cu 2p_1/2_) along with lower-intensity components at 932.6 eV and 952.5 eV corresponding to Cu^+^ species. The coexistence of mixed copper valence states together with strong metal-ligand interactions supports the redox-active nature and surface stability of the nanoparticles (**Fig. S3**). The apparent difference between the XPS and SAED results arises from the different probing depths of these techniques. XPS is inherently surface-sensitive and therefore predominantly detects Cu^2+^ together with a minor Cu^+^ contribution associated with surface oxidation and coordination to the polymethyldopa matrix. In contrast, SAED probes the crystalline interior of the nanoparticles where the diffraction pattern reflects the crystal structure of the nanoparticle core. Accordingly, the combined results are consistent with a predominantly crystalline Cu core surrounded by a thin oxidized Cu-containing surface layer a structural feature commonly reported for copper-based nanoparticles exposed to ambient conditions.

The adopted synthesis protocol together with the FTIR, XPS, TGA, XRD, and EDX results are consistent with the formation of Cu-chelated polymethyldopa nanoparticles reported previously for catechol-based polymeric nanozymes. The observed metal-ligand interactions, changes in the ligand vibrational bands mixed Cu oxidation states revealed by XPS and the presence of a stable organic matrix collectively support the successful formation of the PMD-Cu hybrid nanostructure.

Following confirmation of the PMD-Cu hybrid nanostructure, the combined characterization results further demonstrate that the synthesized nanoparticles possess excellent physicochemical properties. TGA confirms their high thermal stability while DLS, SEM, and TEM reveal a relatively uniform particle size distribution (50–80 nm) and good morphological homogeneity. The polycrystalline nature of the nanoparticles is evidenced by the SAED pattern whereas XPS confirms the coexistence of Cu^2+^ and Cu^+^ species arising from surface oxidation and strong metal-ligand interactions. Collectively, these findings demonstrate that the prepared PMD-Cu nanoparticles exhibit excellent structural integrity, colloidal stability, and redox-active characteristics making them attractive candidates for catalytic, sensing, and bioelectrochemical applications.

### Evaluation of laccase-like catalysis by PMD-Cu NPs

**Fig. 3:**
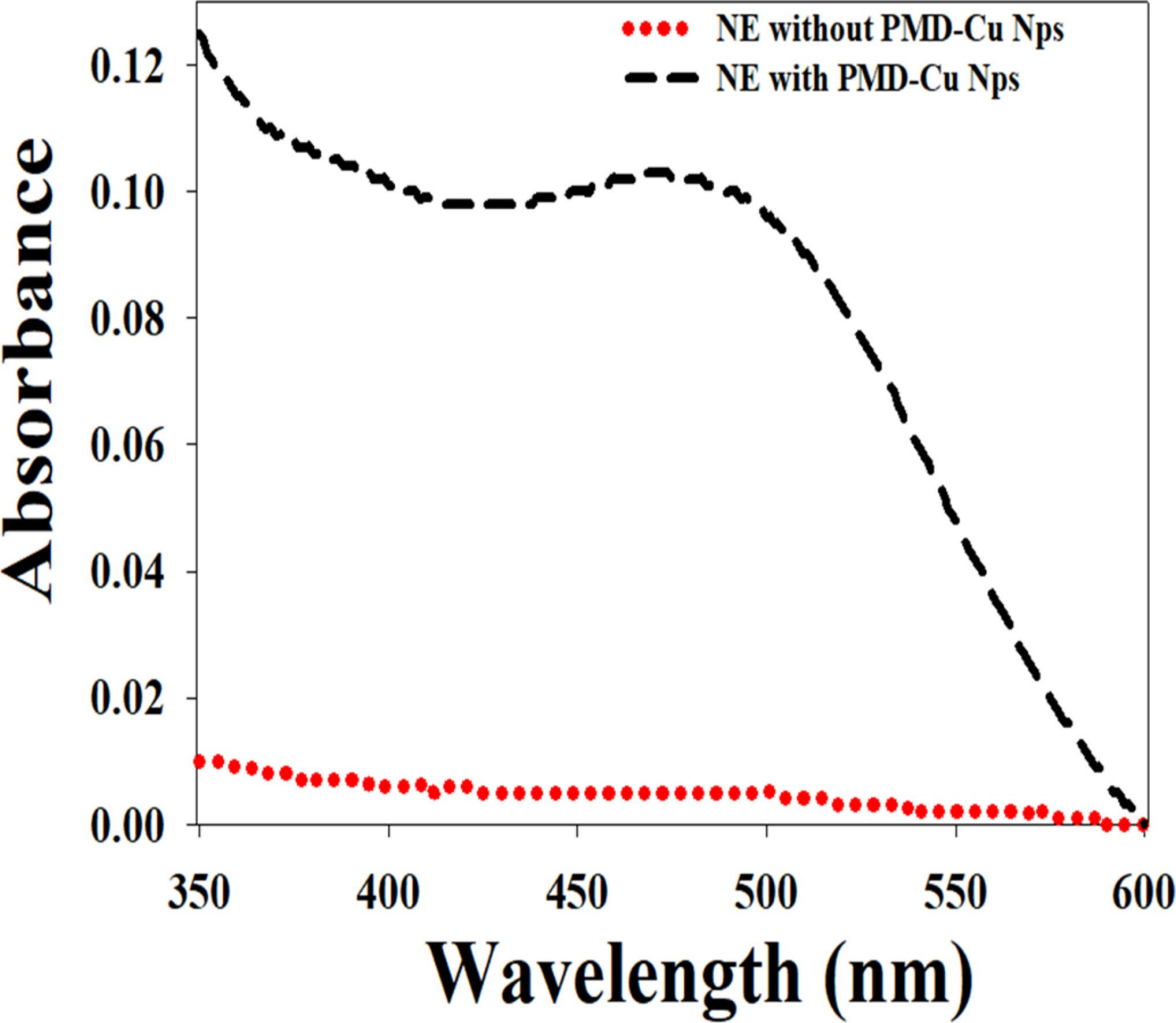
Investigation of PMD-Cu NPs catalytic activity on oxidation of Norepinephrine.

**Fig. 4:**
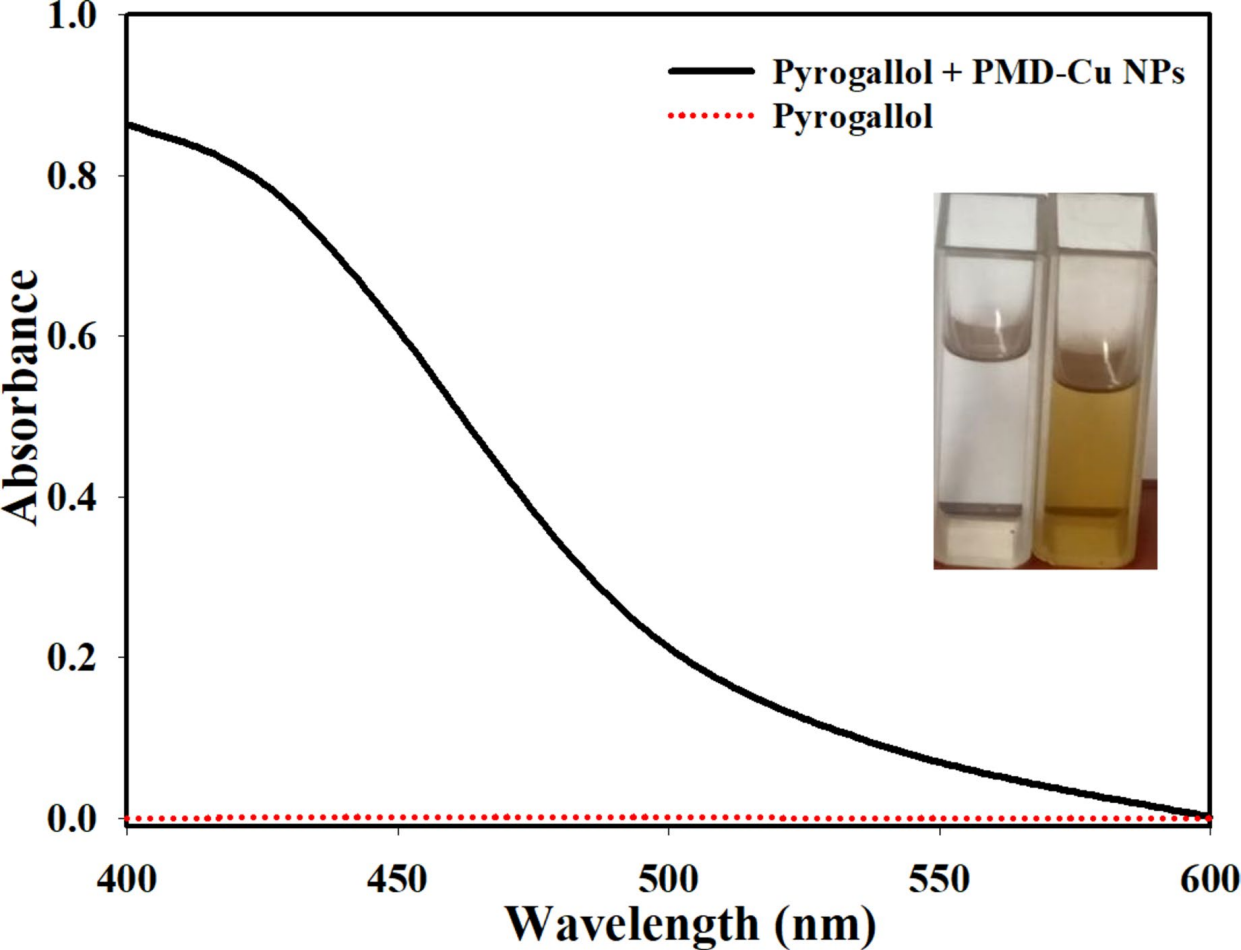
UV-Vis absorption spectra of pyrogallol and its oxidation product generated in the presence of PMD-Cu NPs showing a characteristic absorption maximum at 420.0 nm. Inset: Photographs of the reaction solution before (left) and after (right) oxidation demonstrating the formation of the yellow-colored oxidation product.

**Fig. 5:**
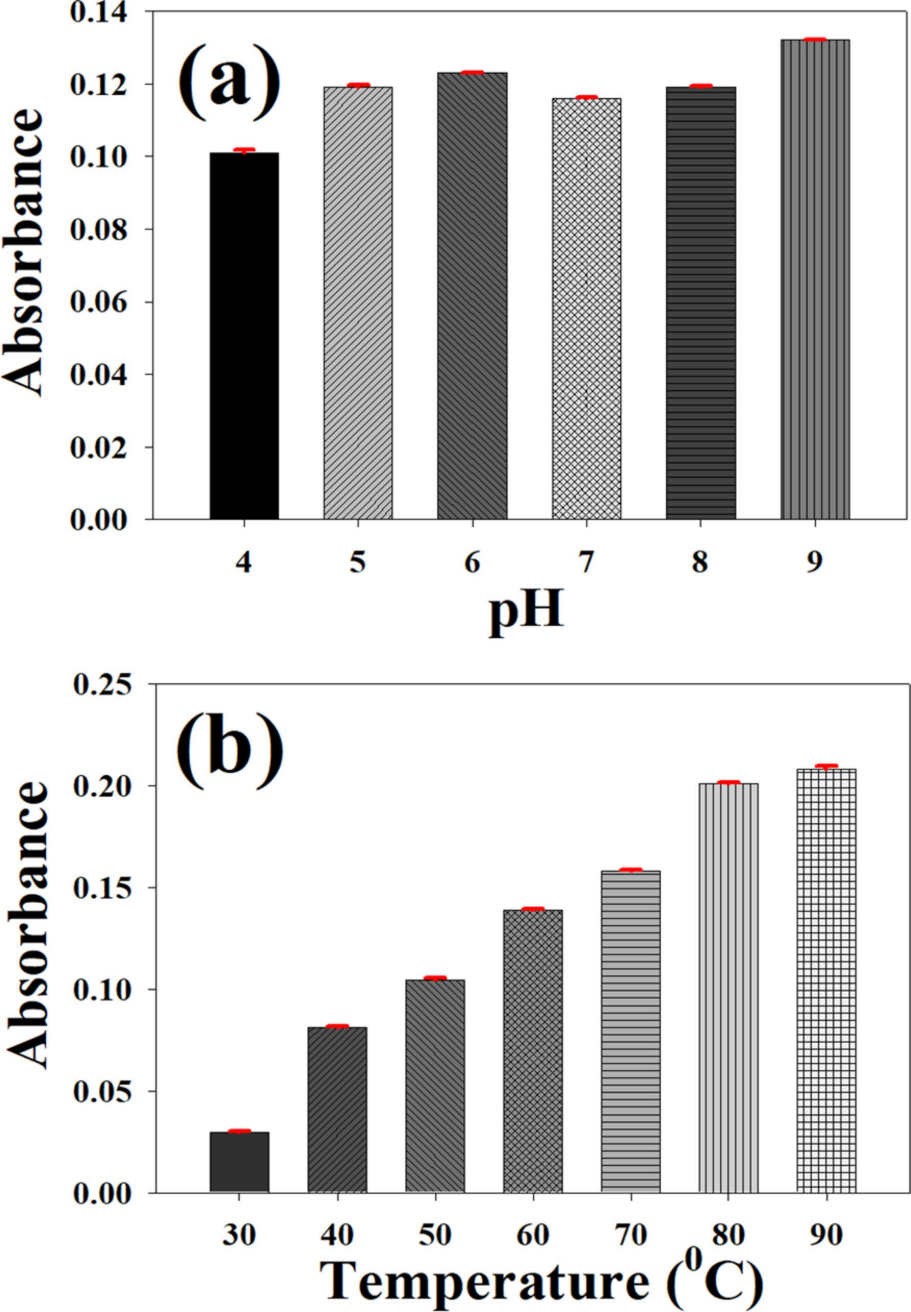
Influence of pH (**a**) and temperature (**b**) on the catalytic performance of PMD-Cu NPs toward norepinephrine oxidation. Data are presented as the mean ± standard deviation (SD) from three independent experiments (*n*= 3).

The Laccase-like activity of PMD-Cu NPs was evaluated using norepinephrine (NE) as a model substrate due to its sensitivity to oxidative conversion^48,49^. As shown in Fig. 3, NE incubated with PMD-Cu NPs exhibited a pronounced absorbance at 485.0 nm consistent with oxidation to norepinephrine-o-quinone. In contrast, NE alone displayed negligible spectral changes confirming minimal auto-oxidation by dissolved oxygen. The laccase-like activity of PMD-Cu NPs was further tested using pyrogallol a representative phenolic pollutant and a well-established laccase substrate. Pyrogallol does not absorb in the visible region, however its oxidation product produce a chromogenic product which is yellow in color and absorbs at 420.0 nm; Fig. 4. Therefore, the reaction can easily be monitored using UV-visible spectroscopy. The influence of pH on catalytic performance was examined over a range of 5.0–9.0 (Fig. 5a). Maximum activity was observed at pH 6.0 with decreased oxidation at lower and higher pH values producing a characteristic bell-shaped profile. This behavior is typical of Cu-based fungal laccase-like systems and reflects optimal proton-coupled electron transfer under mildly acidic conditions^24,50,51^. Although higher absorbance was observed at pH 9.0, this response is attributed to spontaneous norepinephrine auto-oxidation under alkaline conditions rather than enhanced catalytic efficiency^52,53^. A control experiments was performed to measure absorbance changes at different pHs in the absence of a catalyst to confirm autooxidation in alkaline conditions **Fig. S4**.

Overall, pH dependence closely resembles that of natural laccase systems. Temperature-dependent activity was evaluated from 30 to 90 °C (Fig. 5b). Oxidation efficiency increased steadily with temperature reaching a maximum between 80 and 90 °C indicating high thermal robustness. For subsequent kinetic and sensing experiments, 40 °C was selected to maintain physiological relevance while retaining sufficient catalytic activity for practical applications^54^.

### Kinetic analysis and detection of norepinephrine

**Fig. 6:**
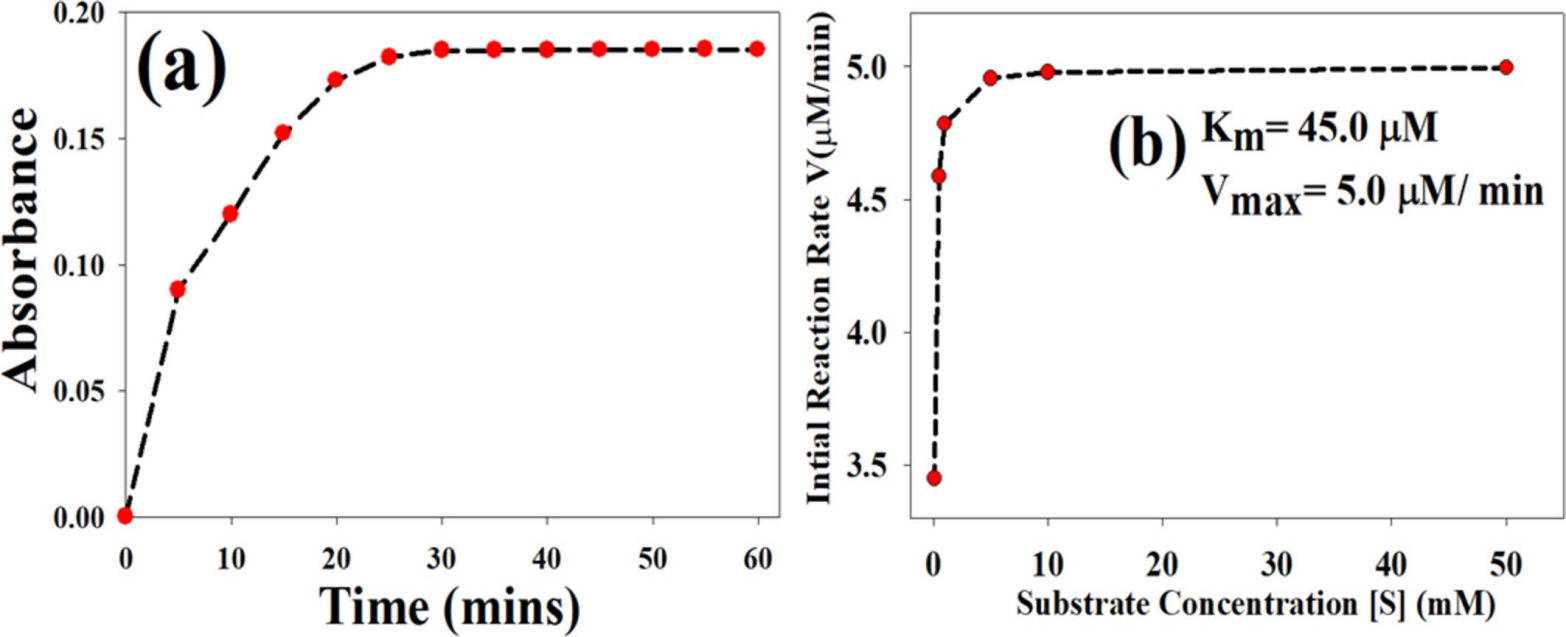
(**a**) Time-dependent oxidation of norepinephrine catalyzed by PMD-Cu NPs under the optimized experimental conditions (phosphate buffer, pH 6.0, 40 °C). (**b**) Michaelis-Menten representation illustrating the relationship between the initial reaction rate and norepinephrine concentration. The apparent kinetic parameters were *K*_*m*_ = 45.0 µM and *V*_*max*_ = 5.0 µM/ min. Data are presented as the mean ± standard deviation (SD) from three independent experiments (*n*= 3).

**Scheme 1:**
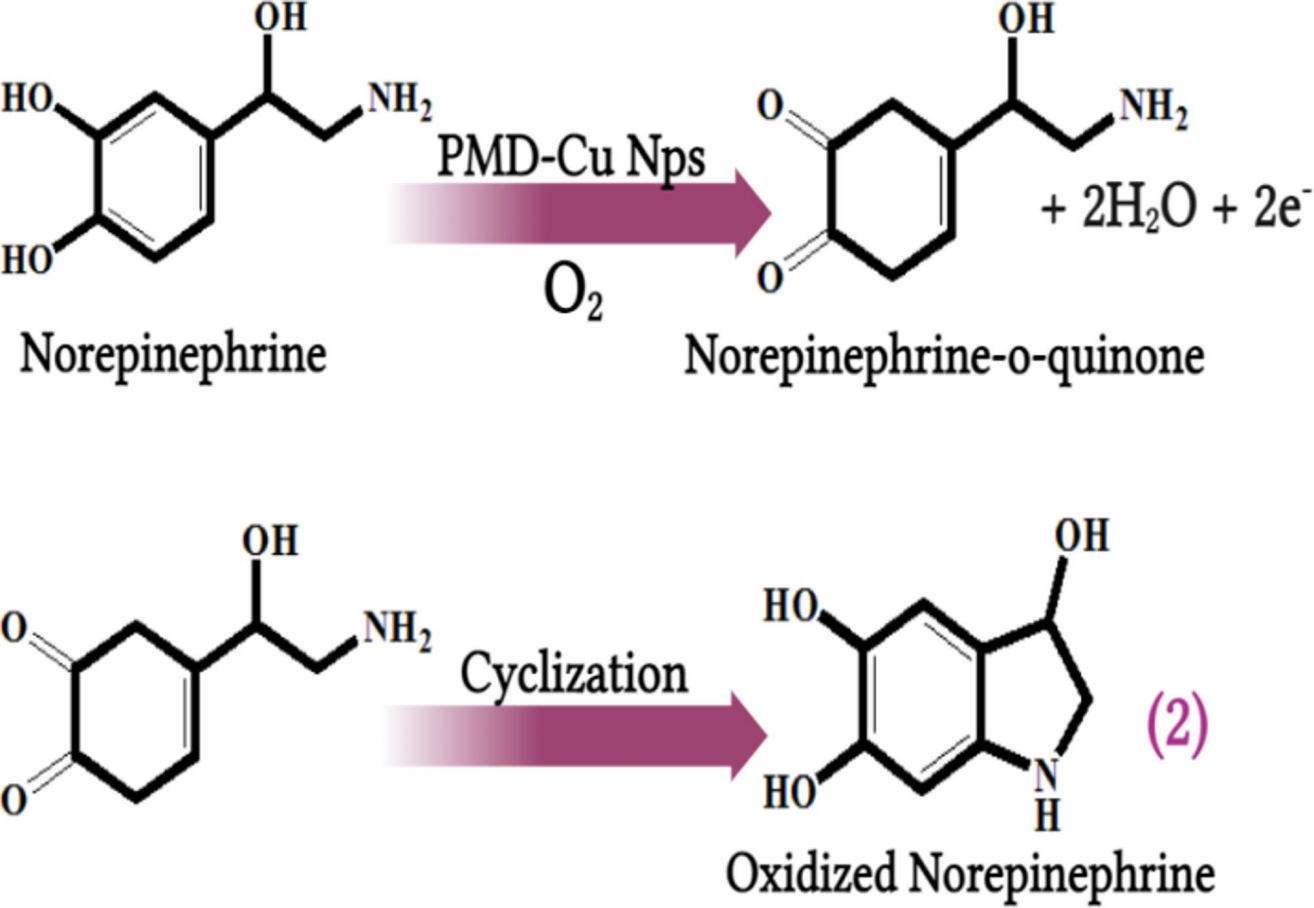
Proposed mechanism of Norepinephrine oxidation by PMD-Cu Nps.

**Fig. 7:**
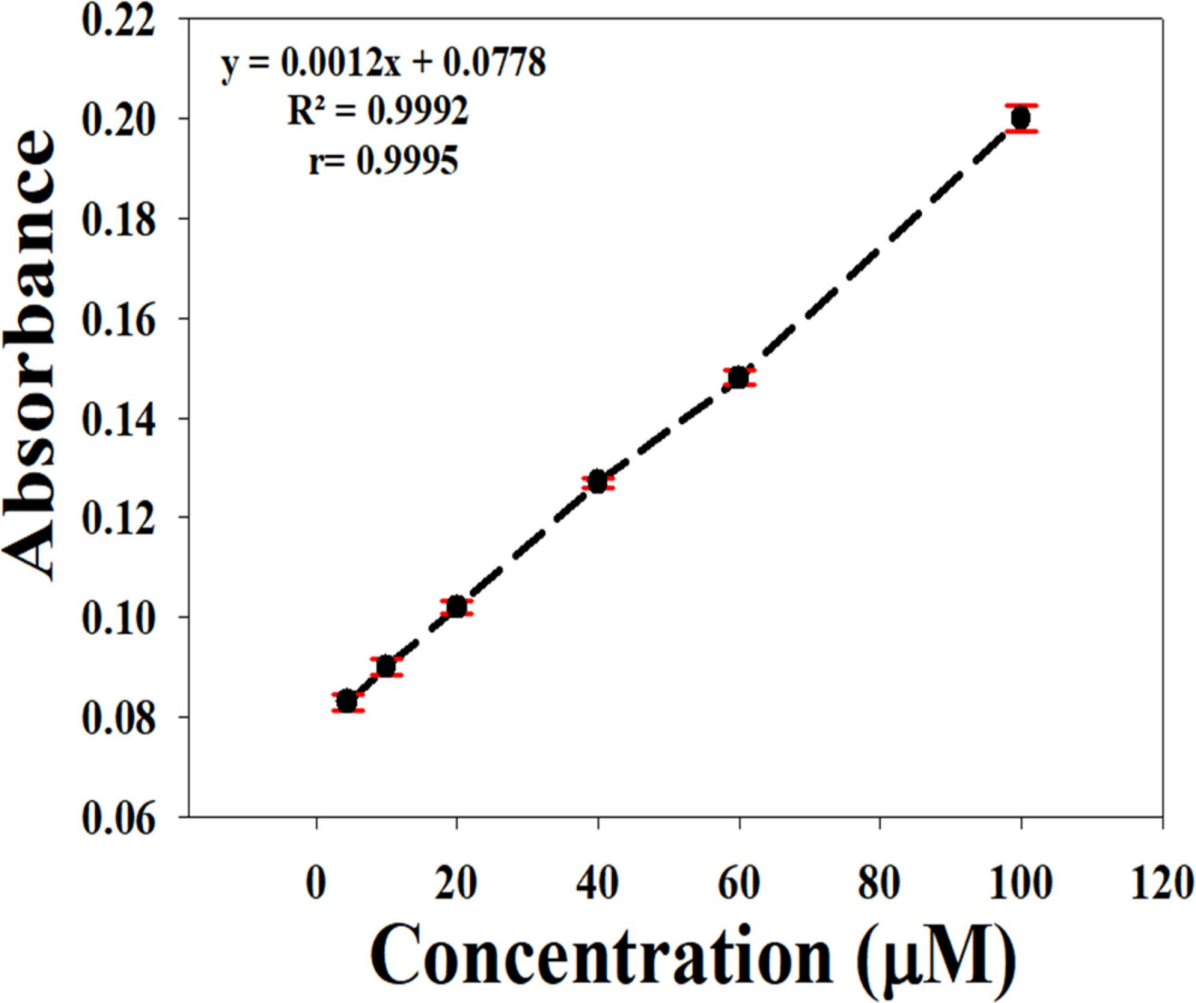
Calibration curve for the colorimetric determination of norepinephrine using PMD-Cu NPs. Data are presented as the mean ± standard deviation (SD) from three independent experiments (*n* = 3).

The time course of NE oxidation was monitored under the optimized experimental conditions using phosphate buffer (pH 6.0) at an incubation temperature of 40 °C. As shown in Fig. 6a, the absorbance increased progressively with reaction time and reached a plateau after approximately 30–35 min indicating substrate depletion or the accumulation of oxidation products. These results confirm the time-dependent conversion of NE to norepinephrine-o-quinone with concurrent release of 2 H^+^ and 2 e^-^, as depicted in Scheme 1. The substrate-dependent catalytic behavior of the PMD-Cu nanozyme was further evaluated using the Michaelis-Menten model as shown in Fig. 6b. The initial reaction rate increased rapidly with increasing norepinephrine concentration before gradually approaching a maximum value exhibiting the characteristic saturation behavior of enzyme-catalyzed reactions. Using a fixed PMD-Cu NP, concentration (100 µg/ mL) and norepinephrine concentrations ranging from 0.1 to 50 mM the apparent kinetic parameters were determined to be *K*_*m*_ = 45.0 µM and *V*_*max*_ = 5.0 µM/ min. The relatively low apparent *K*_*m*_ value indicates a high affinity of the PMD-Cu nanozyme toward norepinephrine while the high *V*_*max*_ demonstrates efficient catalytic turnover. Collectively, these findings confirm the excellent enzyme-like catalytic performance of the PMD-Cu nanozyme and its suitability for sensitive colorimetric sensing applications. PMD-Cu NPs exhibited strong potential for colorimetric sensing of NE. A linear relationship between absorbance and NE concentration was obtained over the range of 4.5–100 µM (Fig. 7) covering physiologically relevant concentrations found in biological fluids^55^. Under optimized conditions, the limits of detection and quantification were determined to be 4.13 µM and 4.85 µM, respectively. The reproducible analytical performance (Table 1) supports the applicability of this system for NE monitoring in clinical and biomedical contexts.

**Table 1:** Assay parameters and method validation for determination of Norepinephrine utilizing PMD-Cu NPs.

Parameters	Norepinephrine
Linearity	4.5–100 µM
Regression line	
– Slope	0.0012
– Intercept	+ 0.0778
– Correlation coefficient (*r*)	0.9995
Accuracy (mean ± SD) ^a^	100.82 ± 0.30
Precision, (% RSD)	
– Repeatability ^b^	0.285
– Intermediate precision ^c^	0.597
LOD^d^	4.13
LOQ^e^	4.85

^a^Average of three determinations (5, 50, and 90 µM).

RSD%^b^, RSD%^c^: the intra-day & inter-day (*n* = 3) relative standard deviation of concentrations (4.5, 40, and 60 µM).

Limit of detection (LOD)^d^ and quantification (LOQ)^e^ were calculated using the following equations: LOD = 3.3 ϭ/S, and LOQ = 10 ϭ/S where “ϭ” is the mean of standard deviation of intercept and “S” is the slope.

The PMD-Cu NPs synthesized in this study exhibit clear laccase-mimicking catalytic behavior as demonstrated by the efficient oxidation of norepinephrine (NE) under ambient aqueous conditions. The visible color change observed in the absence of hydrogen peroxide or additional cofactors confirms their function as oxidase-like nanozymes, a highly desirable feature for environmental remediation and biosensing applications. Unlike natural laccase enzymes, which suffer from narrow operational windows and require strict storage and handling conditions^56^, PMD-Cu NPs maintain high catalytic activity across a broader pH range and elevated temperatures demonstrating their robustness and suitability for real-world deployment. Compared to previously reported copper-based nanozymes, many of which rely on inorganic copper oxides or hybrid MOF architectures, the PMD-Cu system benefits from an organic coordination environment provided by methyldopa. This ligand offers redox-active catechol groups and strong chelation through amino functionalities enhancing both catalytic efficiency and structural stability. Earlier Cu-MOF systems^51^ and Cu-dopamine analogs^50,57^ showed phenolic oxidation activity but were often limited by poor aqueous stability or recyclability. In contrast, PMD-Cu NPs retained consistent catalytic stability over repeated cycles indicating enhanced durability arising from the stable methyldopa-Cu coordination environment. Thermal and pH stability further support their applicability in environmental settings where conditions may fluctuate unpredictably. Whereas natural laccase typically denatures above 60 °C and operates optimally only within a narrow pH range (pH 3–6), PMD-Cu NPs retain substantial activity up to 80 °C and across a wider pH spectrum.

The apparent kinetic parameters further highlight the catalytic efficiency of the developed nanozyme. PMD-Cu NPs exhibited an apparent *K*_*m*_ of 45.0 µM and an apparent *V*_*max*_ of 5.0 µM/ min demonstrating strong substrate affinity together with efficient catalytic activity. Compared with previously reported copper-based nanozymes including the Cu-gallic acid MOF reported by Hussein et al.^24^ and the Cu-MOF catalyst described by Liang et al.^51^ the present PMD-Cu nanozyme provides an attractive balance between substrate affinity and catalytic turnover (Table 2) making it highly suitable for both norepinephrine sensing and catalytic oxidation.

**Table 2:** Kinetic parameters of PMD-Cu NPs compared with other copper-based nanozymes.

Catalyst	*K*_*m*_ (µM)	*V*_*max*_ (µM/min)	References
**Cu-GMP nanozymes**	119.9	82.7	24
**Cu-Gallic Acid**	60.0	4.1	51
**PMD-Cu NPs**	45.0	5.0	This Work

**Fig. 8:**
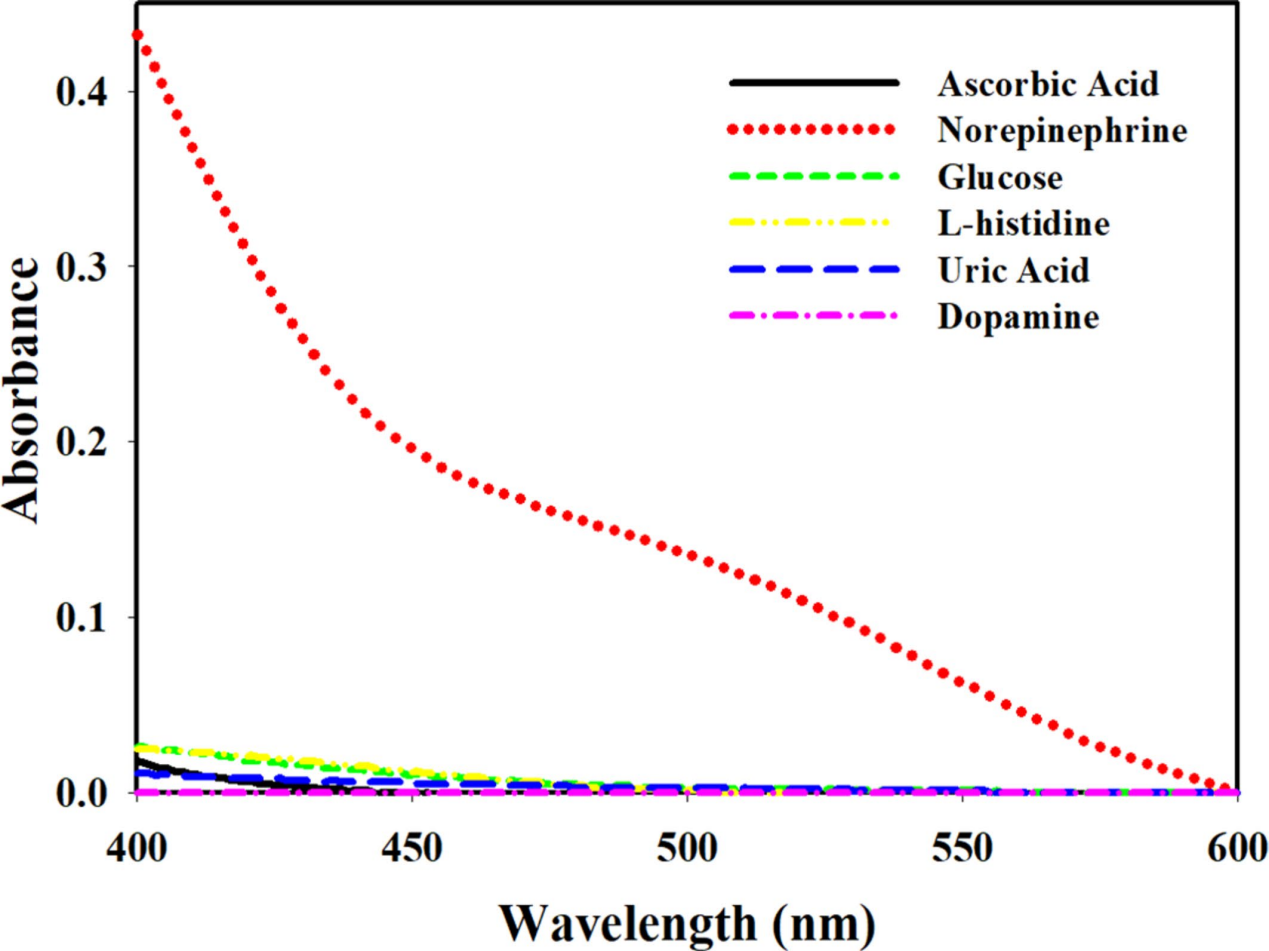
UV-Vis absorption spectra of common potentially interfering substances under identical experimental conditions demonstrating the high selectivity of the PMD-Cu NP-based colorimetric assay. Negligible absorbance responses were observed for the interfering compounds confirming minimal interference.

Further, the specificity of the PMD-Cu NPs for epinephrine oxidation was tested in the presence of common interfering substances dopamine, ascorbic acid, L-histidine, uric acid and glucose. In the presence of 100 µM of the interfering agents, there was no prominent change in the absorption intensity at 485.0 nm (Fig. 8), suggesting that the PMD-Cu NPs can be used for highly sensitive detection of norepinephrine.

### Investigation of PMD-Cu NPs reusability in the oxidative catalysis of NE

The PMD-Cu NPs reusability was evaluated in oxidative catalysis of NE over multiple cycles as shown in **Fig. S5.** The PMD-Cu NPs retained high percentage of its initial catalytic activity after repeated use, across multiple cycles, this demonstrates the superior reusability and structural stability of the PMD-Cu NPs catalyst making it a good option for long-term applications.

### Evaluation of sustainability and analytical performance

**Fig. 9:**
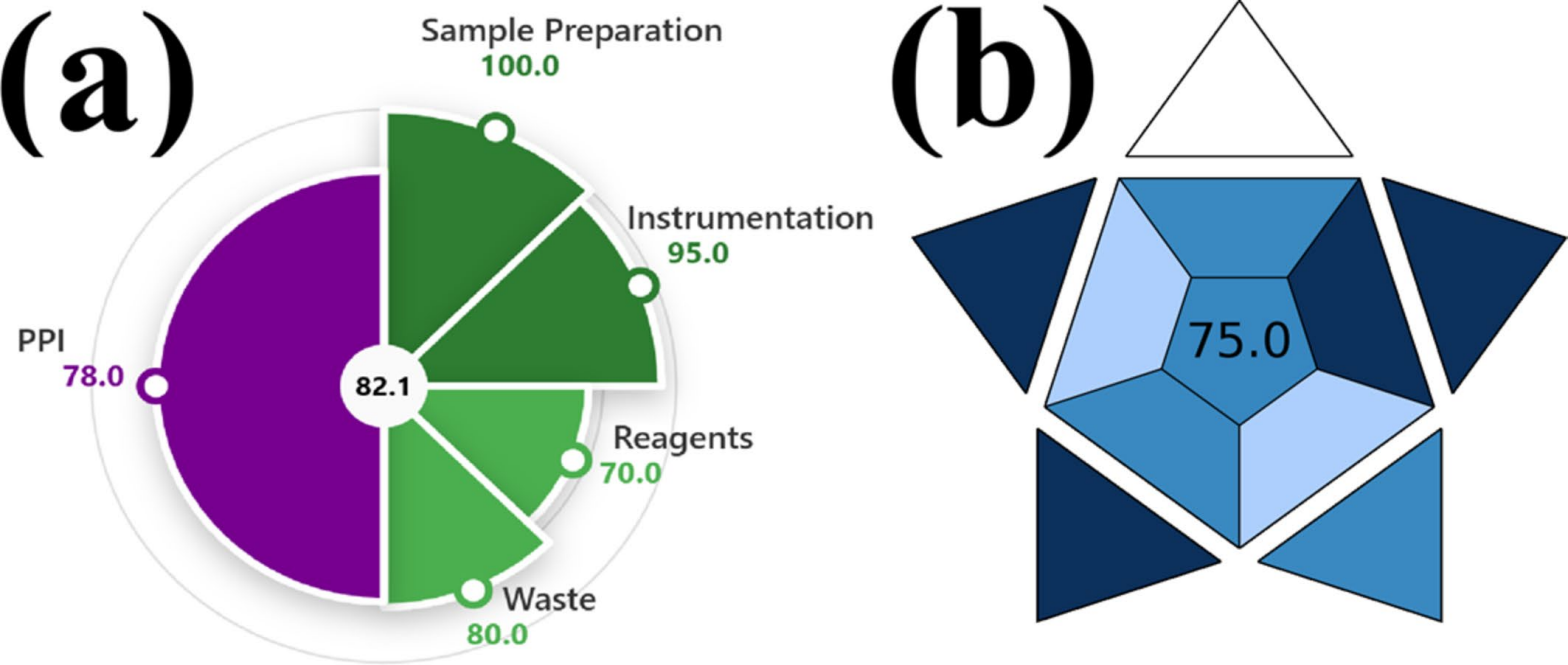
Sustainability and analytical performance assessment of the proposed PMD-Cu NP-based colorimetric sensing platform. (a) Environmental Performance and Practicality Index (EPPI) evaluation illustrating the overall sustainability score together with the individual contributions of sample preparation, instrumentation, reagents, waste generation, and the Practicality Performance Index (PPI). (b) Blue Applicability Grade Index (BAGI) assessment demonstrating the analytical applicability and overall greenness of the developed method with an overall BAGI score of 75.0 confirming excellent practical suitability and compliance with green analytical chemistry principles.

The environmental sustainability and practical applicability of the developed PMD-Cu nanoparticle-based colorimetric sensing platform were comprehensively evaluated using the Environmental Performance and Practicality Index (EPPI)^58,59^ together with the Blue Applicability Grade Index (BAGI)^60^ as presented in Fig. 9. The EPPI assessment (Fig. 9a) yielded an overall score of 82.1 indicating that the proposed analytical strategy provides an excellent balance between environmental sustainability and practical analytical performance^61–67^. The individual category scores demonstrated outstanding performance for sample preparation (100.0) and instrumentation (95.0) reflecting the simplicity of the analytical workflow and the use of readily accessible instrumentation. The method also achieved favorable scores for waste management (80.0), Practicality Performance Index (PPI, 78.0) and reagent consumption (70.0) highlighting the low chemical demand, reduced waste generation, and operational efficiency of the proposed protocol.

The BAGI assessment (Fig. 9b) further confirmed the excellent analytical applicability of the developed method by providing an overall score of 75.0. This favorable rating reflects the use of aqueous media, minimal reagent consumption, environmentally benign experimental procedures, and the avoidance of sophisticated or energy-intensive analytical instrumentation. Collectively, the EPPI and BAGI evaluations demonstrate that the proposed PMD-Cu NP sensing platform successfully integrates analytical sensitivity with green chemistry principles offering a rapid, practical, and environmentally sustainable approach for the colorimetric determination of norepinephrine while minimizing environmental impact.

## Conclusion

In this study, PMD-Cu nanoparticles were successfully synthesized using methyldopa as a multifunctional organic ligand to create a robust laccase-mimicking nanozyme. The methyldopa coordination environment provides redox-active catechol moieties and strong Cu chelation through amino functionalities enabling efficient oxidase-like activity under mild, aqueous, and peroxide-free conditions. The PMD-Cu NPs catalyzed the oxidation of norepinephrine (NE) with a clear and quantifiable colorimetric response demonstrating their dual functionality as both catalytic and sensing platforms. Systematic evaluation of catalytic performance revealed optimal activity at pH 6.0 and 40 °C conditions relevant to biological and environmental systems. Importantly, the nanozyme retained substantial activity across a broad pH range and at temperatures up to 80 °C far exceeding the operational stability of natural laccase enzymes which typically denature above 60 °C and function within narrow pH windows. This enhanced robustness highlights a key advantage of the PMD-Cu system for deployment in complex or fluctuating environments. Kinetic analysis underscores the effectiveness of this nanozyme, yielding a low *K*_*m*_ value of 45.0 µM and a high *V*_*max*_ of 5.0 µM/ min. These parameters reflect strong substrate affinity combined with rapid catalytic turnover and compare favorably with previously reported copper-based nanozymes. The improved kinetic balance is attributed to the enzyme-mimetic copper coordination environment created by methyldopa which promotes efficient electron transfer while maintaining structural stability. From an analytical perspective, PMD-Cu NPs enabled sensitive and reliable colorimetric detection of NE over a concentration range of 4.5–100 µM encompassing physiologically relevant levels found in plasma and urine under both normal and stress conditions. The ability to simultaneously catalyze phenolic oxidation and generate a quantitative optical signal positions this system as a bifunctional platform for combined detection and degradation of phenolic compounds. Overall, the combination of facile synthesis, excellent structural robustness, favorable kinetics performance, and operational versatility establishes PMD-Cu nanoparticles as promising, cost-effective alternatives to natural enzymes. Beyond NE sensing, this work provides a generalizable strategy for designing organic-metal nanozymes with tunable activity and enhanced stability, opening pathways for applications in biosensing, diagnostics, environmental remediation, and green catalytic technologies.

## Supplementary Information

Below is the link to the electronic supplementary material.

Supplementary Material 1

## Data Availability

All data generated or analyzed during this study are included in this published article.
