# Supplementary material for: Synthesis of methyldopa-copper nanoparticles with laccase mimics activity for colorimetric detection of norepinephrine
Source: Sci Rep. 2026 Jul 17;16:22513. doi: 10.1038/s41598-026-61978-6 (PMC13379576; doi:10.1038/s41598-026-61978-6)
Supplement: Supplementary file 1 — Supplementary Material 1 [file 41598_2026_61978_MOESM1_ESM.docx]

**Supplementary Material**

**For**

**Synthesis of Methyldopa-Copper Nanoparticles with Laccase Mimics Activity for Colorimetric Detection of Norepinephrine**

**Aya A. Mouhamed^1*^, Amr M. Mahmoud^1^, Jeffrey G. Bell^2,3^, Ola G. Hussein^4*^**

*^1^Department of Pharmaceutical Analytical Chemistry, Faculty of Pharmacy - Cairo University, Kasr El-Aini Street, ET-11562, Cairo -Egypt*

*^2^Department of Chemistry, Washington State University, Pullman, WA, 99164, United States.*

*^3^The Gene and Linda Voiland School of Chemical Engineering and Bioengineering, Washington State University, Pullman, WA, 99164, Unites States.*

*^4^Department of Pharmaceutical Chemistry, Faculty of Pharmacy, Future University in Egypt, Cairo, 11835, Egypt*

^*^Corresponding authors’ emails: [aya.ahmed@pharma.cu.edu.eg](mailto:aya.ahmed@pharma.cu.edu.eg), [ola.farag@fue.edu.eg](mailto:amr.bekhet@pharma.cu.edu.eg)


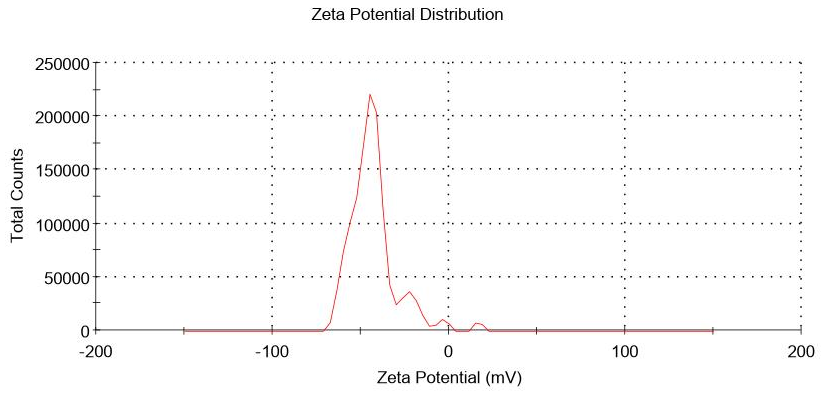


**Fig. S1.** Zeta potential distribution of PMD-Cu nanoparticles dispersed in water demonstrating the surface charge characteristics and colloidal stability of the prepared nanoparticles.

**
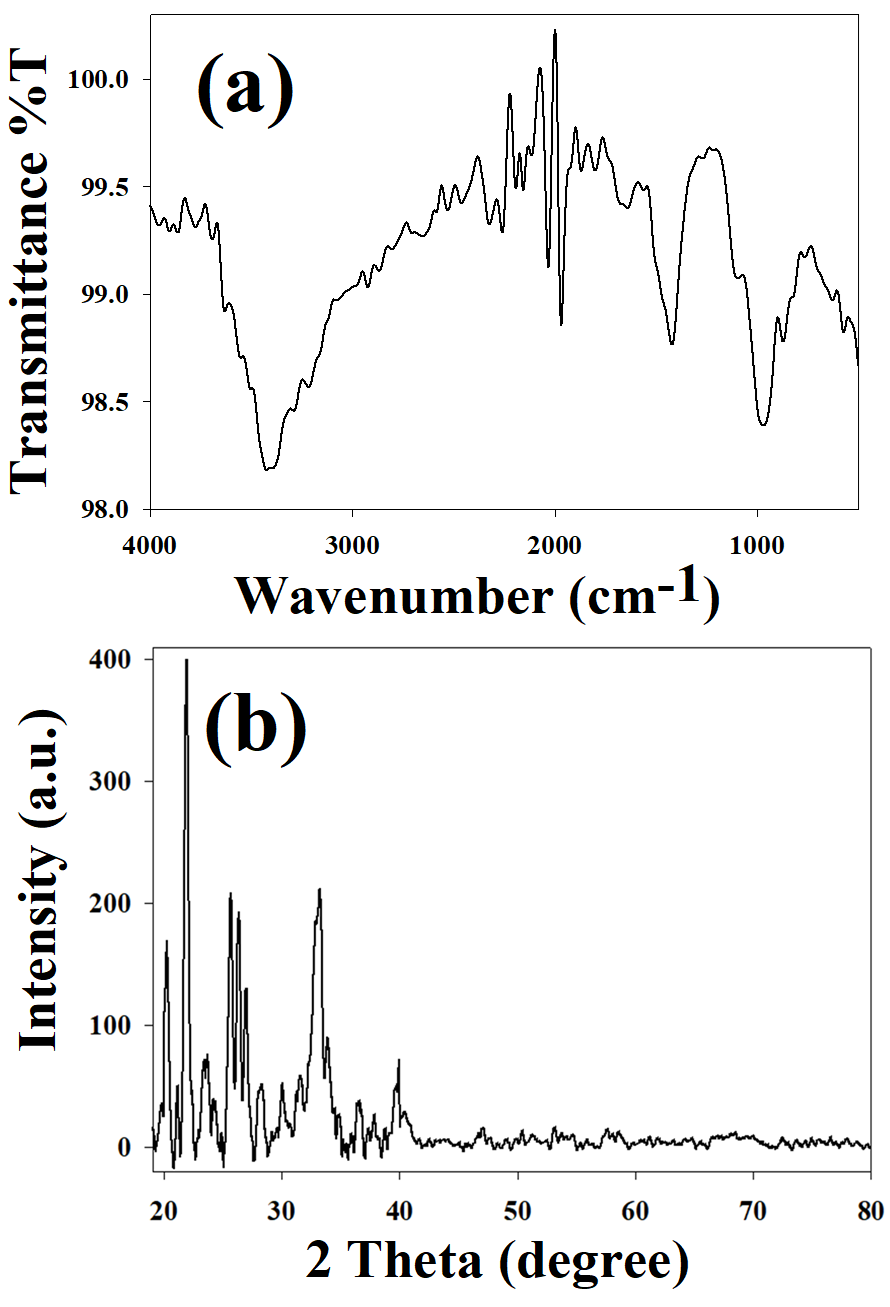
**

**Fig. S2.** (a) FTIR Spectrum and (b) PXRD analysis of PMD-Cu NPs.

**
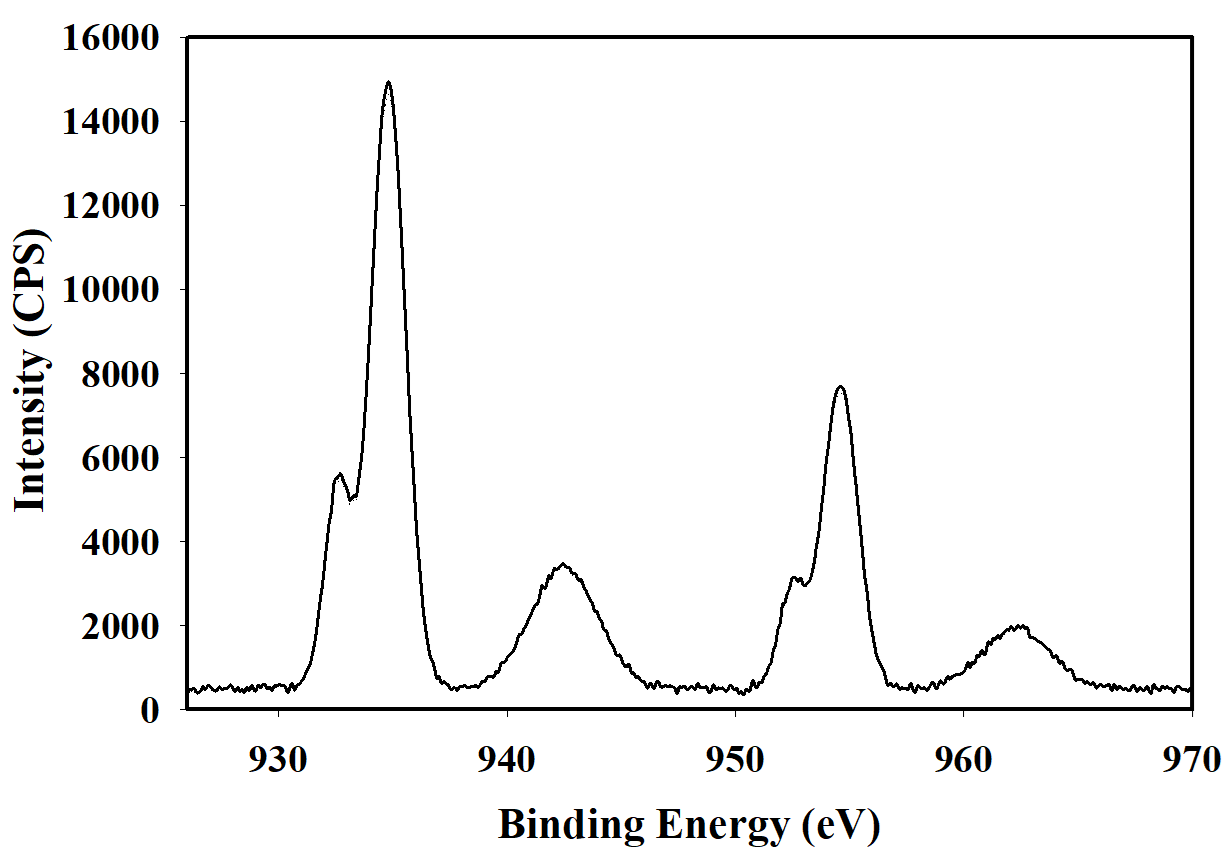
**

**Fig. S3.** High-resolution XPS spectrum of the Cu 2p region for PMD-Cu nanoparticles. Deconvolution of the spectrum reveals dominant Cu²⁺ species with characteristic peaks at 934.8 eV (Cu 2p_3/2_) and 954.6 eV (Cu 2p_1/2_) accompanied by lower-intensity peaks at 932.6 eV and 952.5 eV assigned to Cu^+^ species. The coexistence of Cu^2+^ and Cu^+^ species indicates strong Cu-ligand interactions and the redox-active surface chemistry of the PMD-Cu nanoparticles. As a surface-sensitive technique, XPS primarily reflects the oxidation states of copper at the nanoparticle surface which complement the bulk structural information obtained from SAED.

**
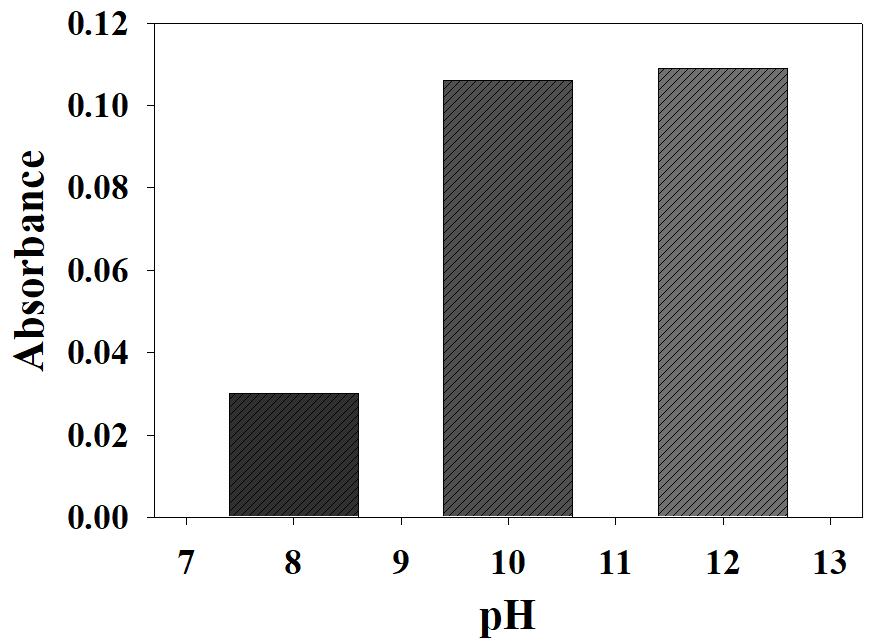
**

**Fig. S4.** Control experiment showing the absorbance of norepinephrine solutions at different pH values in the absence of PMD-Cu NPs. The increased absorbance under alkaline conditions (pH 9-12) confirms spontaneous auto-oxidation of norepinephrine whereas minimal oxidation occurs under near-neutral conditions.


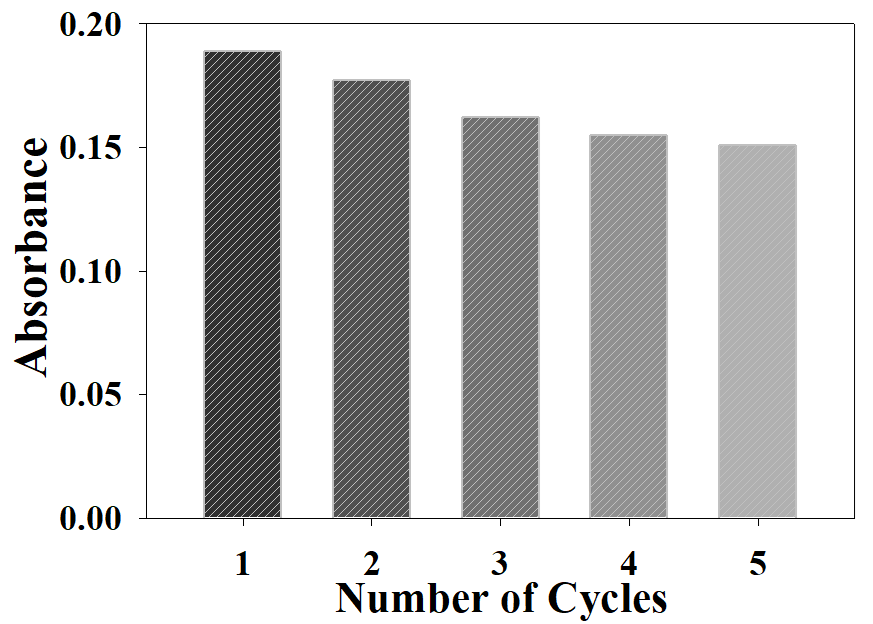


**Fig. S5.** Reusability of PMD-Cu NPs over five consecutive catalytic cycles demonstrating sustained catalytic activity and excellent operational stability during repeated use.
